# Treatment Interruption and Variation in Tablet Taking Behaviour Result in Viral Failure: A Case-Control Study from Cape Town, South Africa

**DOI:** 10.1371/journal.pone.0023088

**Published:** 2011-08-08

**Authors:** Lisa-Noelle Ncaca, Katharina Kranzer, Catherine Orrell

**Affiliations:** 1 Desmond Tutu HIV Foundation, University of Cape Town, Cape Town, South Africa; 2 Faculty of Infectious and Tropical Diseases, London School of Hygiene and Tropical Medicine, London, United Kingdom; University of Cape Town, South Africa

## Abstract

**Background:**

Understanding of the impact of non-structured treatment interruption (TI) and variation in tablet-taking on failure of first-line antiretroviral therapy (ART) is limited in a resource-poor setting.

**Methods:**

A retrospective matched case-control analysis. Individuals failing ART were matched by time on ART with 4 controls. Viral load (VL) and CD4 count were completed 4-monthly. Adherence percentages, from tablet returns, were calculated 4-monthly (interval) and from ART start (cumulative). Variation between intervals and TI (>27 days off ART) were recorded. Conditional multivariate logistic regression analysis was performed to estimate the effect of cumulative adherence <90%, at least one episode of adherence variation >10% and TI on virological failure. Age, gender, baseline log VL and CD4 were included as possible confounders in the multivariate model.

**Results:**

244 patients (44 cases, 200 controls) were included. Median age was 32 years (IQR28–37), baseline CD4 108 cells/mm3 (IQR56–151), VL 4.82 log (IQR4.48–5.23). 94% (96% controls, 86% failures) had cumulative adherence >90%. The odds of failure increased 3 times (aOR 3.01, 95%CI 0.81–11.21) in individuals with cumulative adherence <90%, 2.2 times (aOR 2.20, 95%CI 1.04–4.64) in individuals with at least one episode of fluctuating adherence of >10% and 4.01 times (aOR 4.01, 95%CI 1.45–11.10) in individuals with TIs. For individuals with TI and cumulative adherence >95%, the odds of failing were 5.65 (CI 1.40–22.85).

**Conclusion:**

It is well known that poor cumulative adherence increases risk of virological failure, but less well understood that TI and variations in tablet-taking also play a key role, despite otherwise excellent adherence.

## Introduction

Whilst the connection between adherence and virological outcomes is well established, there remains more to be understood about how patterns of adherence shape those outcomes [1]. As antiretroviral treatments have developed, so has the understanding about how much adherence is required for treatment success. [2]–[4]. Much effort has been put into patient education and adherence programs to help patients attain high adherence targets. Despite this, patient adherence is still often characterised by missed doses, fluctuation in adherence and treatment interruptions (TIs), the impact of which needs to be understood in order to provide good clinical care. [5]–[7] Missed doses and TIs have been a recent topic of interest in the literature, but limited studies, particularly from sub-Saharan Africa, have examined the impact on virological outcome [5], [8], [9].

Treatment interruptions may be structured or non-structured. Structured treatment interruptions, or provider-guided alternating periods of being on and off treatment, are harmful and are not recommended [10]–[12]. Non-structured treatment interruptions are unplanned, and the period without exposure to antiretroviral therapy varies. They may be initiated by a doctor e.g. due to toxicity or poor adherence, or by a patient e.g. due to pill fatigue or a side effect. These treatment interruptions are the reality of patient and doctor behavior although they are likely to cause harm. TIs are known to predict drug resistance [8], disease progression [13], [14], viral rebound [15], and failure [16]. As more is understood about the type and impact of TIs, the more attuned the interventions to prevent these breaks in therapy may become.

This retrospective, case-control study examines the impact of poor or varying tablet taking behavior (when in possession of ART) and lack of drug exposure through a treatment interruption on the risk of failure of first-line antiretroviral therapy in a resource-poor ART clinic in Cape Town, South Africa.

## Methods

### Setting

The study was conducted at the Hannan-Crusaid Treatment Centre (HCTC) in the Klipfontein health sub-district, Cape Town, South Africa. This is a predominately low-income urban community that is home to an estimated 420 000 people in mid-2010 with an ante-natal HIV prevalence rate of 24% noted in 2009.[17] The HCTC has provided antiretroviral therapy (ART) for paediatric and adult patients since September 2002.

Patients at the HCTC were serviced by a multi-disciplinary team and ART was dispensed according to the South African National ART guidelines [18]. All patients were commenced on a non-nucleoside reverse transcriptase inhibitor (NNRTI)-based regimen, usually with stavudine (d4T) and lamivudine (3TC). Patients attended at least twice for scheduled visits prior to commencing ART and then at weeks 0, 4, 8, and 16 on treatment, with regular 4-monthly follow-up thereafter. CD4 count and HIV RNA [using the branch DNA hybridisation technique Bayer HIV-1 RNA 3.0 assay (branch DNA)] were completed before starting ART and every 16 weeks thereafter. Virologic failure was defined as two consecutive viral load values >1,000 copies/mL, with a subsequent switch to second line treatment. Safety tests were also conducted according to the South African National ART guidelines [18].

All patients were required to attend a three-session treatment preparedness class, with at least two sessions to be completed before ART was commenced. Each patient was encouraged to disclose to someone in their social support network, to assist them with treatment adherence. At the first clinic visit a peer counsellor was assigned to each patient. The counsellor provided education support, home visits, and liaised with the clinical team. At least one home visit was conducted pre-ART and another in the first 4 weeks on ART. Further home visits were conducted as necessary.

At 4-monthly scheduled visits, and every 4 to 8 weeks in between, tablet returns were counted and an adherence percentage calculated. These were typically done by the peer counsellors with review by the clinicians. Any decrease in adherence to <85%, or any on-treatment viral load >50 copies/ml, triggered a stepped-up adherence intervention including an individual tailored adherence counselling session, provision and instruction on use of a pill box, monthly home visits and the reduction of the ART dispensing window to one month only. This intensive intervention continued until adherence percentage improved and viral load was again <50 copies/ml.

### Study Population and Design

ART naïve adults (>15 years) starting ART at the Hannan-Crusaid Treatment Centre between September 2002 and February 2007 were eligible for this study. A retrospective matched case-control analysis was performed. Cases included patients experiencing virological failure (2 consecutive viral loads >1000 copies/ml). Time to failure was time from initiation of ART to date of confirmatory viral load >1000 copies/ml. Controls were selected randomly from all non-failure patients in the cohort, and were matched to the cases by time on ART. One case was matched to 4–5 controls to form a unit.

Demographic data and laboratory measurements (viral load and CD4 counts) were extracted from the HCTC electronic patient database. This access database is updated weekly and validated every 3 months. Tablet return data (date of visit, number of tablets returned per medication and number of tablets dispensed at the visit) were copied from each patients clinic record and adherence percentages were recalculated for each patient by the study team, using the formula below.
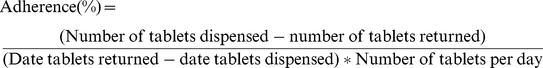



### Adherence Measures and Definitions

**Figure 1:**
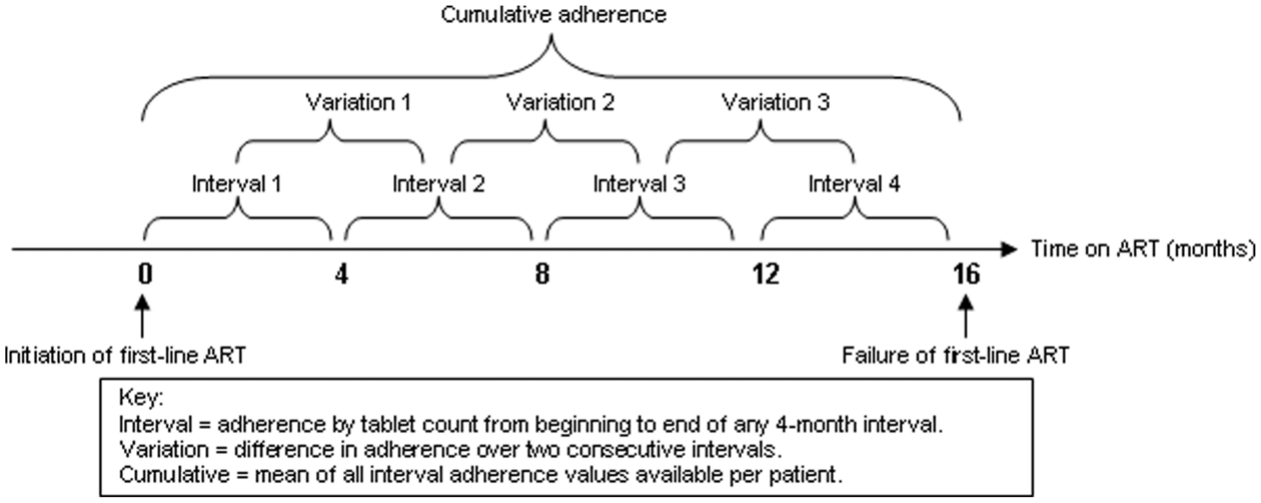
Schematic depicting different adherence measures used.

*Interval adherence* calculated patient's adherence in 4-month intervals i.e. from one scheduled visit to the next using the formula above [Figure 1]. One patient may have multiple interval adherence percentages e.g. weeks 0–16, 16–32, 32–48, etc. Data must have been available for at least 28 days of a 16 week period for an interval adherence percentage to be generated. Interval adherence excluded time when patients were off treatment, i.e. periods of treatment interruption, and gave an estimate of tablet-taking behaviour while in possession of ART.

On occasions when tablet return data was missing due to absent pharmacy records but it was clear from the clinical notes that the patient was still in care and taking medication, the mean of the adherence for the remaining weeks of the interval was used.

*Cumulative adherence* captured the mean adherence from start of ART to point of failure or matched duration of ART for controls [Figure 1]. This was calculated as a mean on the interval adherence values available for each patient. This measure averaged out adherence over the total time in question but excluded time when the patients were off treatment due to treatment interruptions. Each patient only has one cumulative adherence percentage. For the purposes of analysis the adherence percentages were treated as categorical variables. Cumulative adherence was analysed as ≥95% vs. <95%; and again as ≥90% vs. <90%.

*Variation in adherence* was the absolute value of the difference between one interval adherence percentage and that of the subsequent interval. A patient would have multiple adherence variation percentages [figure 1], but were categorised into those with at least one difference between consecutive adherence intervals of ≥10%, or not, for analysis purposes.

*Treatment interruptions* were defined as any period of time off treatment for 28 days or more whether initiated by clinical staff or by the patient. Treatment interruption was categorized into either “no TI” or “one or more TI”. For sensitivity analysis treatment interruptions were further categorized into patient initiated treatment interruptions and doctor initiated interruptions. The latter were further classified into TIs due to toxicity or not.

### Statistical Analysis

Conditional logistic regression analysis was conducted using Stata (version 11, College Station, Texas). Univariate analysis was performed investigating the effect of cumulative adherence <95%, of cumulative adherence <90%, at least one variation of adherence of more than 10% and treatment interruption. Multivariate conditional logistic regression models were used to estimate odds ratios adjusting for potential confounders defined a priori using the following variables: age, gender, baseline viral load and baseline CD4 cell count. Sensitivity analyses were conducted excluding treatment interruptions due to toxicity and doctor initiated treatment interruptions. The effect of treatment interruption was further investigated while adjusting for cumulative adherence.

### Ethics

The University of Cape Town Ethics Committee has approved collection and collation of clinical data collected routinely at the Hannan Crusaid Treatment Centre. Every patient entering the clinic signs an informed consent document to this end.

## Results

### Patient Sample

By February 2007, 2,871 patients had started first-line ART at the Hannan Crusaid Treatment Centre. Only 59 patients had experienced virologic failure at this point.

Of the 59 patients who failed treatment, 44 met eligibility requirements (naïve adults >15 years). These 44 cases were matched to 200 controls. The majority were female (n = 180, 73.8%) and median age at initiation of treatment was 32 years (IQR 28–37), see Table 1. Most patients had symptomatic HIV disease and were commencing ART with at low CD4 counts), see table 1. Median time to failure was 64 weeks (IQR 48–88 weeks). All patients were commenced on NNRTI based regimens, with three patients switched to a regimen containing a protease inhibitor (lopinavir/r) due to NNRTI toxicity. Only 26 of 953 intervals contained missing data (2.7%). 72% of the missing tablet count data occurred due to a period of extreme staff shortage from March to November 2004.

**Table 1:** Baseline patient characteristics at time of ART initiation.

Patients (n)	Total population (n = 244)	Controls (n = 200)	Failures (n = 44)	p-values
**Age: years**	32 (28–37)	32 (28–38)	31 (27–34)	0.0642
**Female: n (%)**	180 (73.8%)	147 (73.5%)	33 (75.0%)	0.838
**WHO Stage: n (%)**				
** Stage III**	127 (52.1%)	107 (53.5%)	20 (45.5%)	0.343
** Stage IV**	67 (27.5%)	51 (25.5%)	16 (36.4%)	
**CD4 cell count (cells/µL)**	108 (56–152)	115 (69–156)	49 (15–103)	<0.001
**HIV-1 Viral Load, log_10_ copies/mL**	4.82 (4.48–5.23)	4.78 (4.43–5.22)	5.00 (4.68–5.52)	0.041

Values are given as median (IQR), unless otherwise stated.

Median cumulative adherence was 97.8% for controls and 96.6% for failures. The majority (81.9%) of all study patients had a cumulative adherence ≥ 95%, whilst only 0.8% had a cumulative adherence below 80%, see table 2. Ninety-six percent of controls had adherence ≥90%, compared to 86.3% of the failures.

**Table 2:** Cumulative adherence presented as proportion of patients per adherence category.

Cumulative Adherence	Total population (n = 244)	Controls (n = 200)	Failures (n = 44)	p-values*
**<80%**	2 (0.8%)	1 (0.5%)	1 (2.3%)	0.088
**80%-<90%**	12 (4.9%)	7 (3.5%)	5 (11.4%)	
**90%-<95%**	30 (12.3%)	24 (12.0%)	6 (13.6%)	
**≥95%**	200 (81.9%)	168 (84.0%)	32 (72.7%)	

Data is presented as number of patients per category with percentage i.e. n (%).

*chi square test for differences in proportions.

Of the 244 patients, 21 (8.6%) interrupted treatment at least once, and there were 23 treatment interruptions in total. The median length of interruption was 79 days (IQR 47–137). There was a median of 186 days (IQR 63–309) from the end of the TI to confirmed virological failure. The majority of the patient-initiated treatment interruptions were related to travelling away from home for an extended period or to non-disclosure of HIV status to those at home. Doctor-initiated treatment interruptions were largely due to drug-related toxicity, or extremely poor adherence noted on pill count (<75%).

### Association between adherence variables and failure

Univariate analyses showed that a cumulative adherence of <95%, cumulative adherence of <90%, at least one episode of adherence variation of ≥10% and at least one episode of treatment interruption increased the odds of failure by 2.09 (95%CI 0.93–4.70), 4.50 (1.31–15.48), 2.07 (95%CI 1.01–4.24) and 4.42 (1.67–11.70) respectively, see table 3. Adjustment for potential confounders revealed similar results. A reduction of cumulative adherence to <90% increased the odds of failing by three times, (adjusted odds ratio (aOR) 3.01 (95%CI 0.81–11.21), whereas at least one episode of variation of >10% resulted in a two times increased odds of failing (aOR 2.20 (95%CI 1.04–4.64) (table 3). The adjusted OR for treatment interruption was 4.01 (95%CI 1.45–11.10). The effect estimate did not change when treatment interruption due to toxicity or doctor initiated treatment interruptions were excluded. Furthermore, adjusting for all potential confounders and cumulative adherence <95% resulted in an aOR of 3.24 (95% 1.03–10.20) for treatment interruption. For a person who had a treatment interruption and a cumulative adherence >95%, the odds of failing treatment were 5.65 (CI 1.40–22.85). The effect of a treatment interruption in individuals with cumulative adherence <95% could not be determined as there were very few individuals with cumulative adherence <95%.

**Table 3:** Multivariate logistic regression analysis: association between adherence variables and failure.

Variable	Univariate OR(95% CI)	Mulitvariate OR (95% CI)*
Cumulative adherence of <95%	2.09 (0.93–4.70)	1.69 (0.71–4.02)
Cumulative adherence of <90%	4.50 (1.31–15.48)	3.01 (0.81–11.21)
One episode of adherence variation of >10%	2.07 (1.01–4.24)	2.20 (1.04–4.64)
≥1 episodes of treatment interruption (≥28 days)	4.42 (1.67–11.70)	4.01 (1.45–11.10)
≥1 episodes of treatment interruption (≥28 days) not due to toxicity	5.62 (1.76–17.97)	3.90 (1.09–13.92)
≥1 episodes of patient initiated treatment interruption (≥28 days)	7.32 (1.30–41.14)	4.40 (0.78–24.93)

*Adjusted for age, sex, baseline CD4 count, baseline viral load.

## Discussion

While it is well-known that poor adherence to ART is key to treatment failure, it is less well understood that treatment interruptions may play as important a role as tablet-taking behaviour. Parienti et al found that treatment interruptions for NNRTIs at low to moderate levels of adherence pose a greater risk of viral rebound than the same number of interspersed missed doses. [1] This study confirms findings of others outside of and within the African context [9], [16], that treatment interruptions can lead to virologic failure.

The new information gained from this study is that the above results hold even when controlling for suboptimal adherence. Thus even patients who keep their cumulative adherence above 95% may not recover from a single treatment interruption of >28days, and proceed to fail treatment. Treatment interruptions are thus not simply a proxy for poor adherence and may reflect a group of people with behaviour that differs from those who are generally poor at taking treatment. Rather than having a chaotic lifestyle e.g. due to alcohol abuse that results in poor adherence, treatment interrupters may be generally good at taking treatment but may experience a single event that results in a treatment interruption. Both behaviours may result in virological failure.

The mean cumulative adherence in this cohort was high for both cases (97.8%) and controls (96.6%), as the ART programme at this site is focussed on adherence support.[19] Failure despite excellent recorded adherence has been noted elsewhere in sub-Saharan Africa. [20] When stratified the failures were less adherent than controls, with 73% of failures having excellent cumulative adherence (>95%), compared to 84% of controls. This creates a unique group of patients to study, as despite intense adherence education, monitoring, intervention, and acceptable levels of cumulative adherence, we still find individuals who fail therapy. Treatment interruptions and variations on adherence may explain some of this, as discussed below, and the use of tablet counts, which typically over-estimate adherence, may also contribute. However further adherence and pharmacokinetic work is needed to explore individuals with discordant adherence and virological response as treatment scale-up continues [21].

Due to the national protocol for naïve patients, all started on a NNRTI-based therapy. The implications of treatment interruption must be explored with this in mind. NNRTI-based therapy has been found to have acceptable levels of suppression at moderate rates of adherence (80%–95%) [2], [3], [22], with resistance usually occurring at lower levels of adherence in contrast to protease inhibitor-based therapy where peak resistance occurs at levels closer to optimal adherence of >95%. [16] However, abrupt treatment interruption of NNRTI therapy can be damaging as the half-life of the NNRTIs is usually longer than that of the accompanying NRTIs resulting in an unprotected NNRTI tail. [9], [23]. This may provide explanation as to why failure occurred in interrupters with otherwise excellent adherence.

Adherence fluctuations of >10% were also shown to have a significant impact on virologic outcomes. Variation was an absolute difference in adherence by tablet count from one quarter to the next, and may have occurred despite adequate adherence during that interval, for example, 93% adherence in one interval and 103% in the next. This may be due to shorter periods of treatment interruption (<28days) that were not captured in this study, but which might have resulted in unprotected exposure of the NNRTI.

The current literature does not have a standard definition for the amount of time off treatment that constitutes a treatment interruption. Different studies use different periods of time, from 3 days to 3 months off treatment.[5]–[7], [24] One of the limitations of this study is that we were not able to capture short intervals of off-treatment time due to our dependence on monthly dispensing records for collection of tablet return and dispensed data. The intention therefore was to capture a significant time off treatment, rather than a few missed doses; however, the significant impact of variation in adherence on treatment outcomes suggests that even missing a few doses may be relevant to virologic outcomes. Some of the intervals had missing data, but only a small proportion (2.7%), and most occurred during a single 6 month period of staff shortage. Thus most of the missing data was missing at random. We also had insufficient power to examine effect modification of TI at different cumulative adherence strata, so, although we know that TIs have an effect in people with excellent adherence, we cannot look at the effect in those with poor adherence. Another limitation is that tablet counts were the only means used to calculate patient adherence. Some studies suggest multiple measures or a composite measure might be a better way to capture adherence. [25], [26] However, in a resource-constrained busy clinic, tablet counts were the quickest and most affordable way of calculating adherence, and our results show correlation of adherence with failure.

The results of this study have several implications for clinical practice and can provide some simple measures to warn providers of potential failure. Often adherence studies focus on cumulative adherence, which can be complex to calculate during a clinic visit, especially for someone who has been on treatment for some time. Interval adherence (adherence from previous to current visit) is a quick and easy measure for clinical or support staff to calculate and variation from one interval to the next gives warning of potential failure prior to viral load testing. Furthermore, treatment interruption is an event that easily identifies an individual as at risk of failure for the clinical team. This study should alert providers that treatment interruptions must be avoided even in the most adherent patients.
